# Core protein mutations are key determinants of hepatitis B virus replication during the hepatitis B e antigen-negative stage

**DOI:** 10.1099/jgv.0.002303

**Published:** 2026-09-04

**Authors:** Diego Flichman, María Cecilia García, Hugo Norberto Granchetti, Rodolfo Campos, María Mercedes Elizalde

**Affiliations:** 1 Facultad de Medicina, Instituto de Investigaciones Biomédicas en Retrovirus y Sida (INBIRS), CONICET, Universidad de Buenos Aires, Buenos Aires, PC 1121, Argentina; 2 Consejo Nacional de Investigaciones Científicas y Técnicas (CONICET), Buenos Aires, PC 1425, Argentina; 3 Facultad de Farmacia y Bioquímica, Cátedra de Farmacia Clínica, Universidad de Buenos Aires, Buenos Aires, PC 1118, Argentina; 4 Facultad de Farmacia y Bioquímica, Departamento de Microbiología, Inmunología, Biotecnología y Genética, Cátedra de Virología, Universidad de Buenos Aires, Buenos Aires, PC 1118, Argentina

**Keywords:** core protein, HBeAg seroconversion, hepatitis B virus, mutations, viral replication

## Abstract

The natural history of chronic hepatitis B virus (HBV) infection comprises distinct stages resulting from virus–host interactions. A late and pivotal event in this process is hepatitis B e antigen (HBeAg) seroconversion, marked by the abrogation of HBeAg expression, a significant reduction in viral load and the accumulation of mutations throughout the genome, particularly within the Core region. While HBeAg loss is associated with mutations in the basal core promoter and preCore regions, these alone do not account for the decreased viral load observed during this stage. To elucidate the contribution of Core variability to HBV replicative capacity, we engineered replication-competent chimeric genomes by reciprocally exchanging the core gene between a WT clone and three HBeAg-negative patient-derived isolates. These constructs were functionally characterized in Huh-7 cells to assess replication intermediates, antigen expression and viral transcriptional activity. Our findings demonstrate that mutations within the Core protein can either impair or enhance HBV replication, depending on their specific mutational patterns. Importantly, all viral replication intermediates were restored to WT levels when the WT Core protein was introduced into the HBeAg-negative genomes. Strong positive correlations between covalently closed circular DNA (CCC DNA) and other viral markers indicate that the Core protein exerts its regulatory effect primarily through regulation of CCC DNA levels. Notably, the absence of Core expression increased CCC DNA transcriptional activity, supporting a repressive role of the WT Core protein in gene expression. Collectively, these findings highlight the pivotal regulatory role of Core protein mutations in modulating HBV replication dynamics and gene expression during the HBeAg-negative phase and underscore its potential as a promising target for novel antiviral strategies.

## Data Availability

All the data generated during the current study are included in the manuscript.

## Introduction

Hepatitis B virus (HBV) infection remains a major global health burden despite therapeutic improvements and the widespread implementation of vaccination programmes. In 2022, the World Health Organization (WHO) estimated that 254 million individuals were living with chronic HBV infection, with 1.2 million new infections and 1.1 million deaths annually, primarily due to cirrhosis and hepatocellular carcinoma (HCC) [1–3]. Moreover, high rates of underdiagnosis and limited access to treatment – particularly in low-income regions – continue to hinder progress toward the WHO’s 2030 elimination goal [4–6]. Addressing these diagnostic and treatment gaps is paramount for global eradication efforts and mitigating the burden of chronic HBV-related disease.

Chronic HBV infection progresses through four distinct clinical phases, each characterized by distinct patterns of viral replication and host immune response [7]. These stages include: (i) hepatitis B e antigen (HBeAg)-positive chronic infection, typically observed in infants and young children infected perinatally, where an immature immune system fails to effectively clear the virus, leading to high viral loads and minimal liver inflammation; (ii) HBeAg-positive chronic hepatitis, marked by activation of the host immune response, resulting in elevated alanine aminotransferase (ALT) levels, high HBV DNA (>6.0 Log_10_ IU ml^−1^) and liver inflammation, potentially manifesting as fatigue, nausea and jaundice; (iii) HBeAg-negative chronic infection (ENCI), where the virus persists with a partially effective immune response, resulting in low viral loads (<3.3 Log_₁₀_ IU ml^−1^), persistently normal ALT levels, reduced liver inflammation often presenting with mild or no symptoms and HBeAg seroconversion; and (iv) HBeAg-negative chronic hepatitis (ENCH), characterized by abnormal or fluctuating ALT levels, intermediate viral loads (3.3–6.0 Log_10_ IU ml^−1^) and more pronounced liver injury, frequently associated with higher intrahepatic necroinflammatory lesions, which can still cause significant liver damage and risk of progression to cirrhosis and HCC. This phase is often associated with the emergence of HBV variants that fail to express or secrete HBeAg.

The HBV genome consists of four partially overlapping open reading frames (ORFs): S (surface), C (core), P (polymerase) and X. These ORFs encode seven viral proteins, including the hepatitis B core protein, HBeAg, hepatitis B X protein, polymerase and three forms of hepatitis B surface antigen (HBsAg) – small (S), medium (M) and large (L) [8]. Among these, the Core protein, a 183-amino acid homodimer, stands out for its pleiotropic roles throughout the viral life cycle. Within the cytoplasm of hepatocytes, the Core protein self-assembles into a 120-dimer icosahedral capsid, which serves as the protective shell for the viral genome [9]. Beyond its essential structural function, recent studies have highlighted its multifaceted regulatory roles in HBV replication and its intricate interaction with the host immune system, including modulation of viral transcription and interaction with various host proteins [10–12]. As a result, the Core protein has emerged as a promising therapeutic target, offering potential avenues for the development of innovative antiviral strategies [13–15].

Our recent characterization of replicating HBV genomes from patients in the ENCH and ENCI phases has provided a comprehensive understanding of how mutations accumulate across the HBV genome after HBeAg seroconversion. Notably, we observed that the core region exhibited the highest degree of heterogeneity among the viral proteins and demonstrated that HBeAg-negative–associated mutations modulate viral replication, antigen expression and secretion and potentially viral pathogenicity, reflecting the complex and evolving host-virus dynamics that characterize chronic HBV infection [16].

To further elucidate these intricate mechanisms and specifically focus on the role of the Core protein in HBV biology, we generated chimeric genomes by exchanging the core region between a WT clone and HBeAg-negative isolates. We then conducted an in-depth functional characterization of these chimeric constructs, aiming to precisely delineate the impact of Core protein variations on viral fitness, replication and interaction with the host, thereby providing critical insights into the pathogenesis of HBeAg-negative chronic HBV infection.

## Methods

### DNA constructs

pUC19 plasmids containing a subgenotype D1 HBeAg-positive WT full-length HBV genome (WT, accession number: OK106256), an HBeAg-positive core‐deficient genome [Core (-)] and subgenotype D1 HBeAg-negative full-length HBV genomes (P#1: PQ655423, P#2: PQ655424 and P#3: PQ655428) were previously described [16, 17].

Chimeric replicative plasmids were constructed by exchanging the region encoding the Core protein (nt 1820–2628). Specifically, the core region from HBeAg-negative clones (P#1, P#2 and P#3) was inserted into the WT genome, and conversely, the core region from the WT virus was introduced into the HBeAg-negative genomes. For this purpose, the WT and HBeAg-negative plasmids were digested with *NdeI and MfeI* restriction enzymes (New England Biolabs, USA), and the corresponding fragments were exchanged to generate the WT/core P#1–3 and P#1–3/core WT chimeric plasmids. Correct insertion of the fragments in the resulting constructs was confirmed by sequencing.

### Cell culture and transfection

Human hepatoma cell line HuH-7 (JCRB Cell Bank #0403) was grown in Dulbecco’s modified Eagle’s medium (DMEM; Sigma, CA, USA), supplemented with 10% FBS (Sigma, CA, USA), 1 mM non-essential amino acids (GIBCO, Carlsbad, CA, USA), 2 mM l-glutamine (GIBCO, Carlsbad, CA, USA), 0.15% sodium bicarbonate, 100 IU ml^−1^ penicillin and 100 µg ml^−1^ streptomycin. The cells were maintained at 37 °C in a humidified atmosphere containing 5% CO_2_.

Full-length linear HBV DNAs (nt 1821–1820) with sticky ends were used for transfections. Briefly, linear HBV monomers were excised from the pUC19 plasmids by restriction with BspQI (New England Biolabs, USA) at 50 °C. The resulting 3.2 kb fragments were gel-purified, and the DNA was quantified by spectrometry. Transient transfections were carried out using X-tremeGene™ 9 transfection reagent (Roche, Mannheim, Germany), according to the manufacturer’s recommendations.

In all experiments, 0.05 µg of the luciferase reporter vector pGL4.13 (luc2/SV40; Promega, WI, USA) was co-transfected with the linear full-length HBV genomes as a transfection efficiency control. To evaluate the light emission produced by the luciferase activity, 20 µl of cell lysates was mixed with the commercial reagent Luciferase Assay System (Promega, WI, USA), according to the manufacturer’s recommendations, and relative light units were detected. Results were expressed in relative light units.

### Analysis of total HBV DNA and cccDNA

Total HBV DNA and CCC DNA detection were performed by quantitative real-time PCR (qPCR). Seventy-two hours post-transfection, cells were lysed with a buffer containing 50 mM Tris–HCl (pH 8), 10 mM EDTA, 100 mM NaCl, 0.5% SDS and 0.5 mg ml^−1^ proteinase K (Invitrogen, USA) and incubated at 56 °C for 2 h. Nucleic acids were purified by phenol-chloroform extraction and ethanol precipitation. To validate the specificity of the assay, the procedure was challenged using HBV DNA extracted from serum samples with high viral loads (8.2 Log10 IU ml^−1^), as well as cell extracts obtained immediately after transfection in order to evaluate possible amplification of input DNA. Under these conditions, the nonspecific cccDNA detection background was estimated to be ∼1 : 1,500 molecules, which, in our view, does not compromise the integrity of the comparative analyses performed.

The primers used for total HBV DNA detection were as follows: sense (nt 205–226): 5′‐ GCGGKGTKTTTCTTGTTGACAA‐3′, antisense (nt 391–373): 5′‐ AACGCCGCAGACACATCC A ‐3′.

To prevent non-specific amplification of non-covalently closed circular forms of HBV DNA, total DNA was treated with 500 U ml^−1^ T5 exonuclease (New England Biolabs, USA) at 37 °C for 1 h to remove non-supercoiled dsDNA. This was followed by heat inactivation at 95 °C for 5 min and fourfold dilution with distilled water. Selective primers for cccDNA: sense (nt 1551–1570) 5′-GTCTGTTCCTTCTCATCTGC-3′ and antisense (nt 1887–1868) 5′-AGGCACAGCTTGGTGGCTTG-3′ were used for the qPCR. Mitochondrial DNA was analysed as an internal reference to normalized cccDNA levels. The primers for mitochondrial DNA detection were: sense 5′-CCCCACAAACCCCATTACTAAACCCA-3′ and antisense 5′-TTTCATCATGCGGAGATGTTGGATGG-3′.

Serial dilutions of an HBV replication-competent plasmid (pCH-9/3091) were used as quantification standards. Relaxed circular HBV DNA (rcDNA) levels were calculated by subtracting CCC DNA from total HBV DNA.

### Analysis of capsid-associated HBV DNA

Intracellular capsid‐associated HBV DNA was analysed by Southern blot. Briefly, transfected cells were treated with lysis buffer (50 mM Tris/HCl pH 7.5; 100 mM NaCl; 1 mM EDTA; 0.5% NP40) and centrifuged at 12,000 ***g*** for 1 min to remove the nuclei. The cell lysate was treated with DNase I (Roche, Mannheim, Germany) at 37 °C for 40 min, and the reaction was stopped by the addition of EDTA. The supernatant was then incubated with 0.5 mg ml^−1^ proteinase K (Invitrogen, Carlsbad, CA, USA) and 1% SDS at 56 °C for 2 h. Nucleic acids were purified by phenol‐chloroform extraction and ethanol precipitation. DNA was separated on a 1.2% agarose gel and transferred onto a positively charged nylon membrane (Roche, Mannheim, Germany). The DNA was immobilized using an ultraviolet crosslinker and hybridized with a digoxigenin (DIG)‐labelled subgenomic probe (Roche, Mannheim, Germany). Hybridization signals were detected using an enzyme‐linked immunoassay (DIG‐High Prime DNA Labelling and Detection Starter Kit II; Roche, Mannheim, Germany).

Extracellular capsid‐associated HBV DNA was quantified by qPCR. Cell culture supernatants were harvested and clarified by centrifugation at 3,000 ***g*** for 10 min. Supernatants were treated with DNase I (Roche, Mannheim, Germany), and nucleic acids were purified as described above. The following primers, that specifically amplify rcDNA, were used for the amplification: sense (nt 1608–1627) 5′‐ATGGAGACCACCGTGAACGC‐3′ and antisense (nt 1887–1868) 5′‐AGGCACAGCTTGGTGGCTTG‐3′. Serial dilutions of an HBV replication‐competent plasmid (pCH‐9/3091) were used as quantification standards.

### Analysis of HBV pregenomic RNA

Total RNAs were extracted with TRIzol reagent (Invitrogen, Carlsbad, CA, USA), according to the manufacturer’s recommendations, treated with DNase I (Roche, Mannheim, Germany) and reverse‐transcribed using random primers. The levels of HBV pregenomic RNA (pgRNA) were detected by qPCR using a TaqMan^®^ probe method, as previously described. The primers and probe used to detect the HBV 3.5 kb RNA were as follows: sense (nt 2295–2312): 5′‐ATAGACCACCAAATGCCC‐3′, antisense (nt 2436–2415): 5′‐CGAGATTGAGATCTTCTGCGAC‐3′ and probe: 5′‐FAM‐CTTATCAACACTTCCGGARACTACTGTTGTTAGAC‐BHQ1‐3′.

### Antigen quantification

The concentration of HBsAg in cell lysates and culture supernatants was measured by electrochemiluminescence immunoassay (ECLIA) using the Elecsys HBsAg II quant II on a Cobas e801 instrument (Roche, Mannheim, Germany). Results were expressed in IU ml^−1^.

### HBV replicative activity

CCC DNA transcriptional activity was estimated by calculating the ratio of pgRNA to CCC DNA molecules (pgRNA/CCC DNA). Virion productivity was defined as the ratio of rcDNA to CCC DNA (rcDNA/CCC DNA). HBsAg productivity was calculated as the amount of extracellular HBsAg per CCC DNA molecule (extracellular HBsAg/CCC DNA).

### Statistical analysis

All experiments were performed independently three times. Statistical significance between samples was determined using analysis of variance followed by post-hoc Tukey’s test. A value of *P*<0.05 was considered to be statistically significant. Results were expressed as mean±sd. All analyses were performed using GraphPad Prism 8 software. Linear correlation between variables was assessed using Pearson’s correlation coefficient, and simple linear regression models were fitted using the least squares method. Cook’s distance was used to detect influential outliers, and the regression results were compared after excluding these values.

## Results

**Fig. 1.**
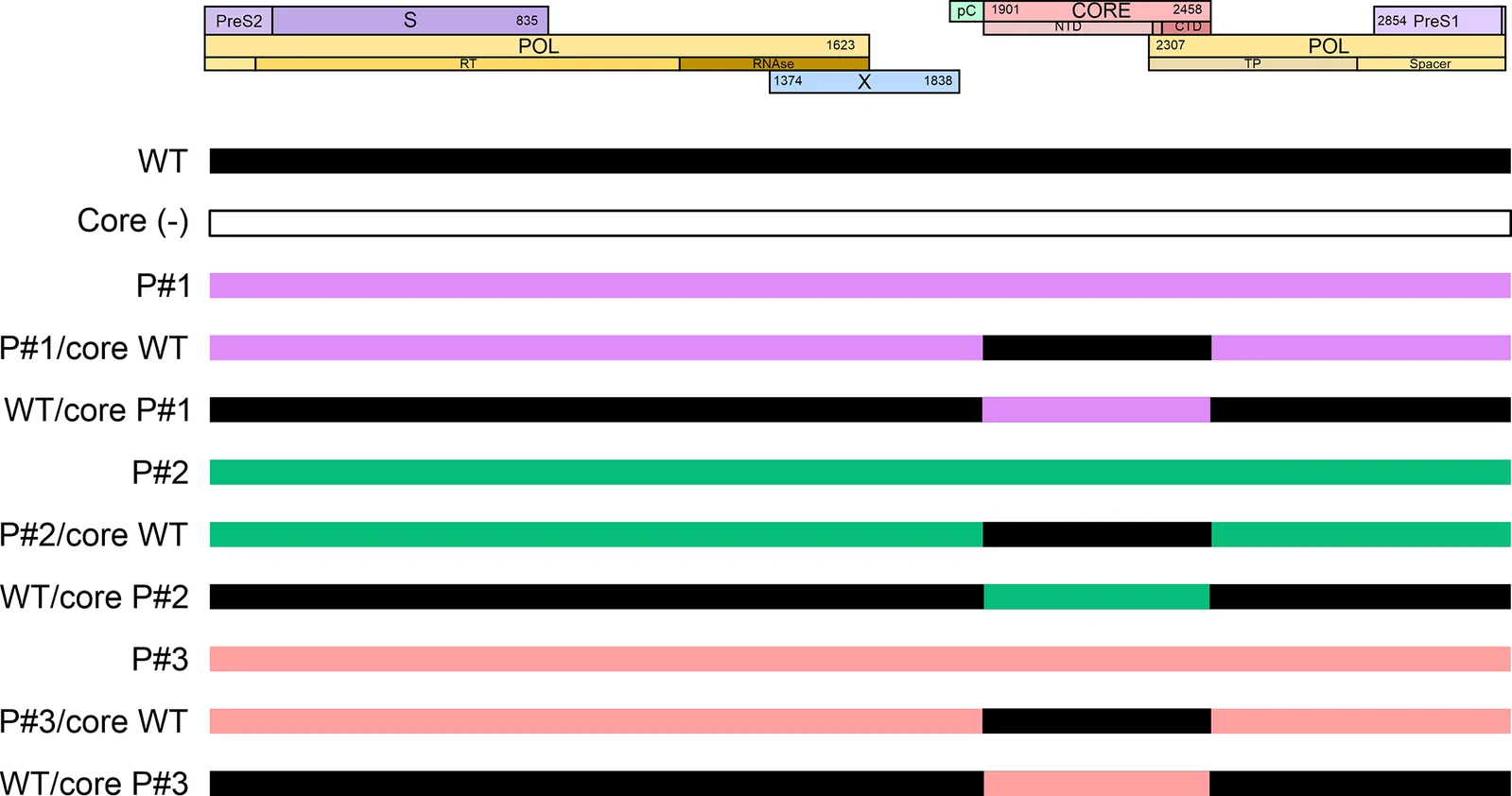
Schematic representation of the HBV chimeric constructs. Chimeric HBV genomes were generated by exchanging the core gene between HBV genomes. Specifically, the core region from three HBeAg-negative isolates (P#1, P#2 and P#3) was individually inserted into an HBeAg-positive WT backbone. Conversely, the core region from the WT genome was introduced into each of the HBeAg-negative genomic backbones.

To investigate the influence of the core region on HBV replicative capacity, a series of chimeric, replication-competent genomes were engineered. Specifically, the Core gene from three distinct HBeAg-negative patient isolates (P#1, P#2 and P#3) was reciprocally exchanged with that of a WT HBeAg-positive genome. This strategy yielded two sets of chimeric constructs: WT genomes containing HBeAg-negative core sequences (WT/core P#1, WT/core P#2, WT/core P#3) and HBeAg-negative genomes carrying the WT core (P#1/core WT, P#2/core WT, P#3/core WT) (Fig. 1). Subsequently, the replicative capacity, antigenic expression and overall viral activity of these engineered constructs were comprehensively evaluated to assess the core region’s specific contributions.

### Analysis of core sequence variability

**Fig. 2.**
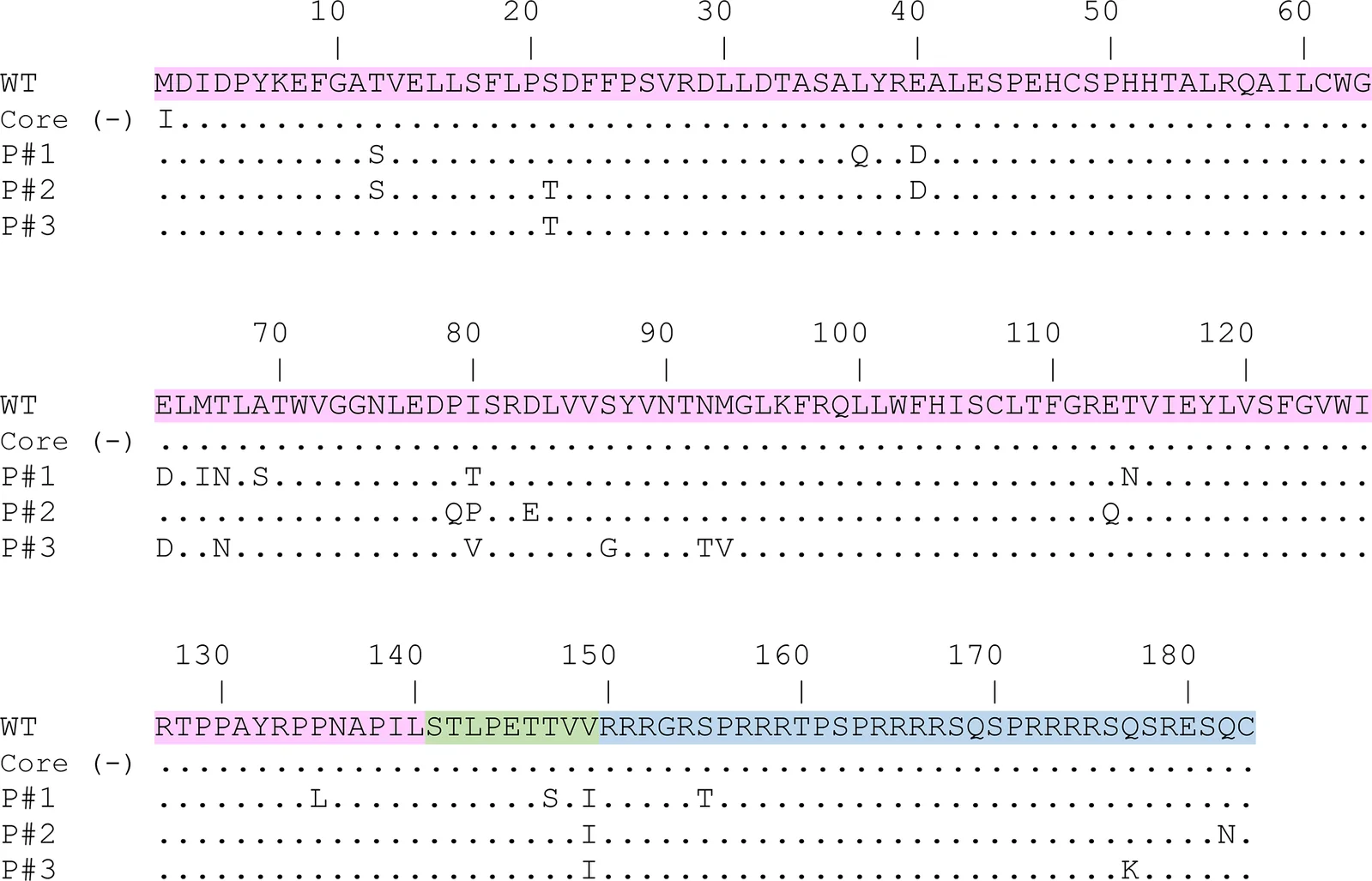
Core sequence variability in the chimeric HBV constructs. Amino acid alignment of the HBV core region is shown for the WT HBV, the core-negative control [Core (-)] and the three HBeAg-negative constructs (P#1, P#2 and P#3). Dots represent identity with the WT sequence, while letters indicate amino acid substitutions. Functional domains of the core protein are highlighted by background colour: N-terminal assembly domain (purple), linker region (green) and arginine-rich C-terminal domain (blue).

Compared to the WT Core gene sequence, constructs P#1, P#2 and P#3 (accession numbers PQ655423, PQ655424 and PQ655428, respectively) exhibited 20, 25 and 17 nucleotide substitutions. Among these, 13 (65%) in P#1, 9 (36%) in P#2 and 9 (52.9%) in P#3 were non-synonymous, leading to amino acid changes. These substitutions were distributed across the three functional domains of the Core protein. Most of the amino acid changes were located within the N-terminal assembly domain (aa 1–140) with 10 substitutions identified in P#1 and 7 in both P#2 and P#3. Additional mutations were also detected in the linker region (aa 141–149), with two substitutions in P#1 and 1 in each of P#2 and P#3, as well as in the arginine‐rich C-terminal domain (aa 150–183), where one substitution was identified in each genome (Fig. 2). Notably, the majority of these substitutions have been previously described as being most frequent in the HBeAg-negative stage [18, 19].

### Replicative capacity

**Fig. 3.**
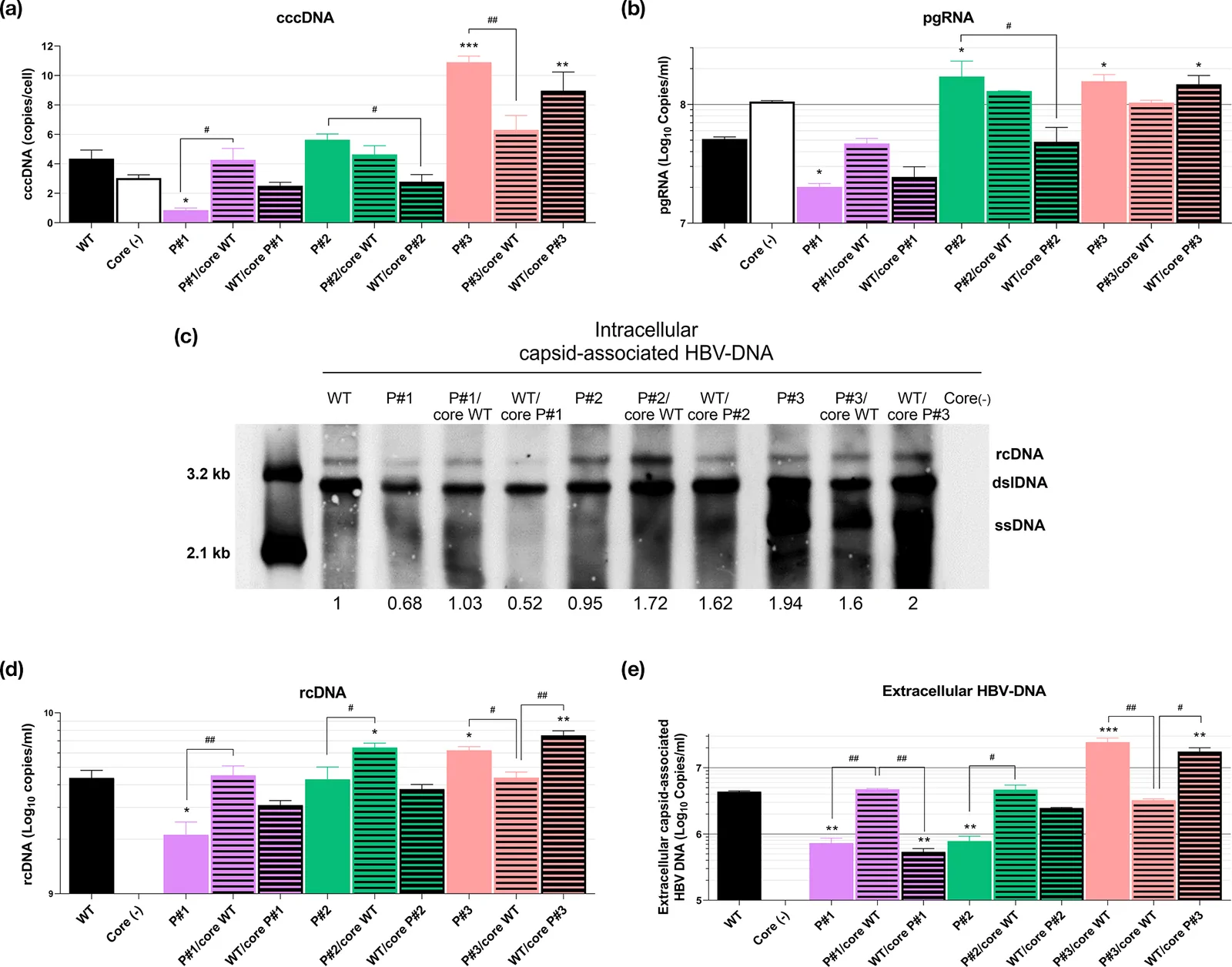
Replicative capacity of the chimeric HBV constructs. Huh‐7 cells were transfected with linear full-length HBV genomes from WT, core-deficient [Core (-)], HBeAg-negative clones (P#1, P#2, P#3) and their respective chimaeras (P#1/core WT, WT/core P#1, P#2/core WT, WT/core P#2, P#3/core WT and WT/core P#3). Cells and supernatants were collected 3 days post-transfection. (a) Total DNA was extracted, treated with T5 exonuclease to remove nonsupercoiled dsDNA, and CCC DNA levels were assessed by qPCR. Results were normalized to mitochondrial DNA. (b) Total RNA was extracted, and pgRNA levels were analysed by RT‐qPCR. (c) Intracellular capsid‐associated HBV DNA was extracted, and replicative intermediates were assessed by Southern blot. The relative amount of ssDNA in relation to WT is shown below the image. (d) Total HBV DNA was quantified by qPCR and rcDNA levels were calculated by subtracting CCC DNA from total HBV DNA. (e) Extracellular capsid‐associated HBV DNA was quantified by qPCR. Shown values represent the mean±sd of three independent experiments. *: significant difference compared to WT; #: significant differences among chimeric constructs. Three symbols: *P*<0.0001; two symbols: *P*<0.005 and one symbol: *P*<0.05. CCC DNA: covalently closed circular DNA; rcDNA: HBV relaxed circular DNA; dsLDNA: HBV double‐stranded linear DNA; ssDNA: HBV single‐stranded DNA; pgRNA: pregenomic RNA.

Analysis of the P#1 chimera construct set revealed that, compared to the WT clone, P#1 showed significantly lower levels of CCC DNA, pgRNA, rcDNA and capsid-associated DNA, both intracellularly and extracellularly. Notably, substitution of the P#1 core with the WT Core gene (P#1/core WT) restored all viral replication intermediates to levels comparable to those of the WT virus. In contrast, incorporation of the P#1 Core gene into the WT backbone (WT/core P#1) resulted in a marked reduction of these viral intermediates to values similar to those observed in the HBeAg-negative P#1 genome (Fig. 3).

For construct P#3, which, unlike P#1, exhibited significantly higher levels of cccDNA, pgRNA, rcDNA and both intracellular and extracellular capsid-associated DNA compared to WT, the opposite effect to that observed in the P#1-derived chimaeras was verified. Specifically, the incorporation of the WT Core gene (P#3/core WT) markedly reduced these viral intermediates to levels comparable with the WT clone. Conversely, the incorporation of the P#3 core gene into the WT genome backbone (WT/core P#3) resulted in a substantial increase in the levels of the viral intermediates, similar to those observed in the original P#3 construct (Fig. 3).

In the case of the P#2 constructs, P#2 showed no difference in CCC DNA, rcDNA and intracellular capsid-associated DNA levels compared to the WT virus. However, it exhibited significantly higher levels of pgRNA and lower levels of extracellular capsid-associated DNA. Upon replacement of the P#2 core gene with the WT core (P#2/core WT), all viral replication intermediates were restored to WT-like levels, except for rcDNA and intracellular capsid-associated DNA, which were significantly increased. In contrast, incorporation of the P#2 core gene into the WT genome (WT/core P#2) did not reproduce the phenotype observed for the P#2 virus: CCC DNA and pgRNA levels were significantly reduced, while both intra- and extracellular capsid-associated DNA levels were significantly increased. Nevertheless, no significant differences were detected between the WT virus and the WT/core P#2 chimeric construct (Fig. 3).

Moreover, the Core (-) construct, which shares the entire nucleotide sequence with the WT virus, except for a point mutation in the start codon of the Core gene that abrogates its synthesis, exhibited a slight increase in pgRNA levels compared to the WT, despite showing no significant differences in cccDNA levels (Fig. 3A and B).

Taken together, these results indicate that incorporation of the WT Core protein into HBeAg-negative genomes restores all viral replication intermediates to levels comparable to those of the WT virus. Furthermore, mutations within the Core protein alone may either enhance or impair viral replication, depending on the specific mutational pattern, highlighting the critical role of the Core protein in modulating viral replicative capacity. In the particular case of P#2 chimeric constructs, the inability of the P#2 core to restore the replicative capacity of the P#2 genome suggests that mutations in other regions of the viral genome may also contribute to the observed replicative phenotype.

### Antigen expression and secretion

**Fig. 4.**
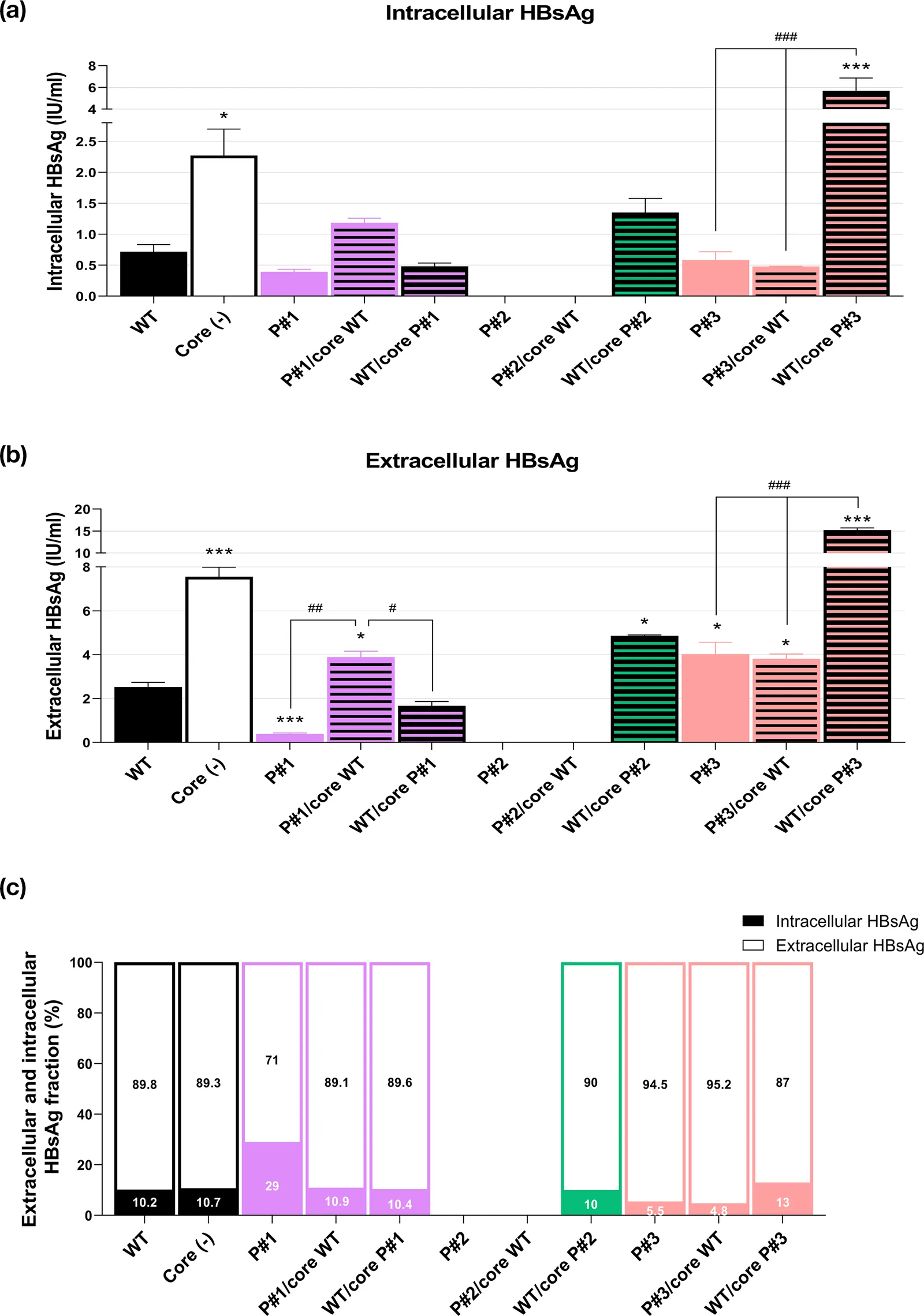
Intracellular and secreted HBsAg levels in chimeric HBV constructs. Huh‐7 cells were transfected with linear full-length HBV genomes from WT, core-deficient [Core (-)], HBeAg-negative clones (P#1, P#2, P#3) and their respective chimaeras (P#1/core WT, WT/core P#1, P#2/core WT, WT/core P#2, P#3/core WT and WT/core P#3). Cells and supernatants were collected 3 days post-transfection. (a) Intracellular and (b) extracellular HBsAg levels were quantified using ECLIA. (c) Extracellular/intracellular HBsAg ratio. Values shown represent the mean±sd of three independent experiments. *: significant difference compared to WT; #: significant differences among chimeric constructs. Three symbols: *P*<0.0001; two symbols: *P*<0.005 and one symbol: *P*<0.05.

In line with the results obtained for replication capacity, the P#1 construct showed reduced expression of HBsAg, both intracellularly and extracellularly, compared to the WT virus. Replacement of the P#1 Core gene with that of the WT virus restored intracellular and extracellular HBsAg levels to those observed in the WT. In contrast, incorporation of the P#1 Core gene into the WT genome reduced HBsAg levels to values comparable to those observed for P#1 (Fig. 4A and B).

Interestingly, for both the P#2 virus and the P#2/core WT chimaera, intra- and extracellular HBsAg levels were below the detection threshold (0.05 IU ml^−1^), suggesting that HBsAg-associated mutations present in the P#2 genome may impair antigen expression or alter epitope recognition by anti-HBs antibodies. Furthermore, the WT/core P#2 chimeric virus showed higher intra- and extracellular HBsAg levels compared to the WT virus (Fig. 4).

On the other hand, for both P#3 and the P#3/core WT chimeric virus, no differences in intracellular HBsAg levels were observed in relation to the WT virus; however, both constructs exhibited increased secretion of HBsAg. In contrast, the WT/core P#3 chimeric virus showed more than a fivefold increase in both intracellular and extracellular HBsAg levels compared to P#3, the P#3/core WT chimaera and the WT virus (Fig. 4a and b).

Furthermore, the Core (-) construct also showed significantly higher levels (∼3.5-fold) of intracellular and extracellular HBsAg compared to the WT virus (Fig. 4a and b).

Finally, analysis of the HBsAg secretion-to-expression ratio among the chimeric constructs revealed that, regardless of the absolute amount of HBsAg produced, constructs in which the S gene derived from the WT genome – such as WT, core (-), WT/core P#1, WT/core P#2 and WT/core P#3 – in addition to P#1/core WT – secreted ∼90% of the expressed antigen. In contrast, P#1 retained ∼30% of the antigen intracellularly, while P#3 and P#3/core WT retained only ∼5% of HBsAg inside the cells (Fig. 4c).

Together, these findings underscore a potential regulatory role of the core protein in modulating HBsAg expression and secretion.

### HBV replicative activity

**Fig. 5.**
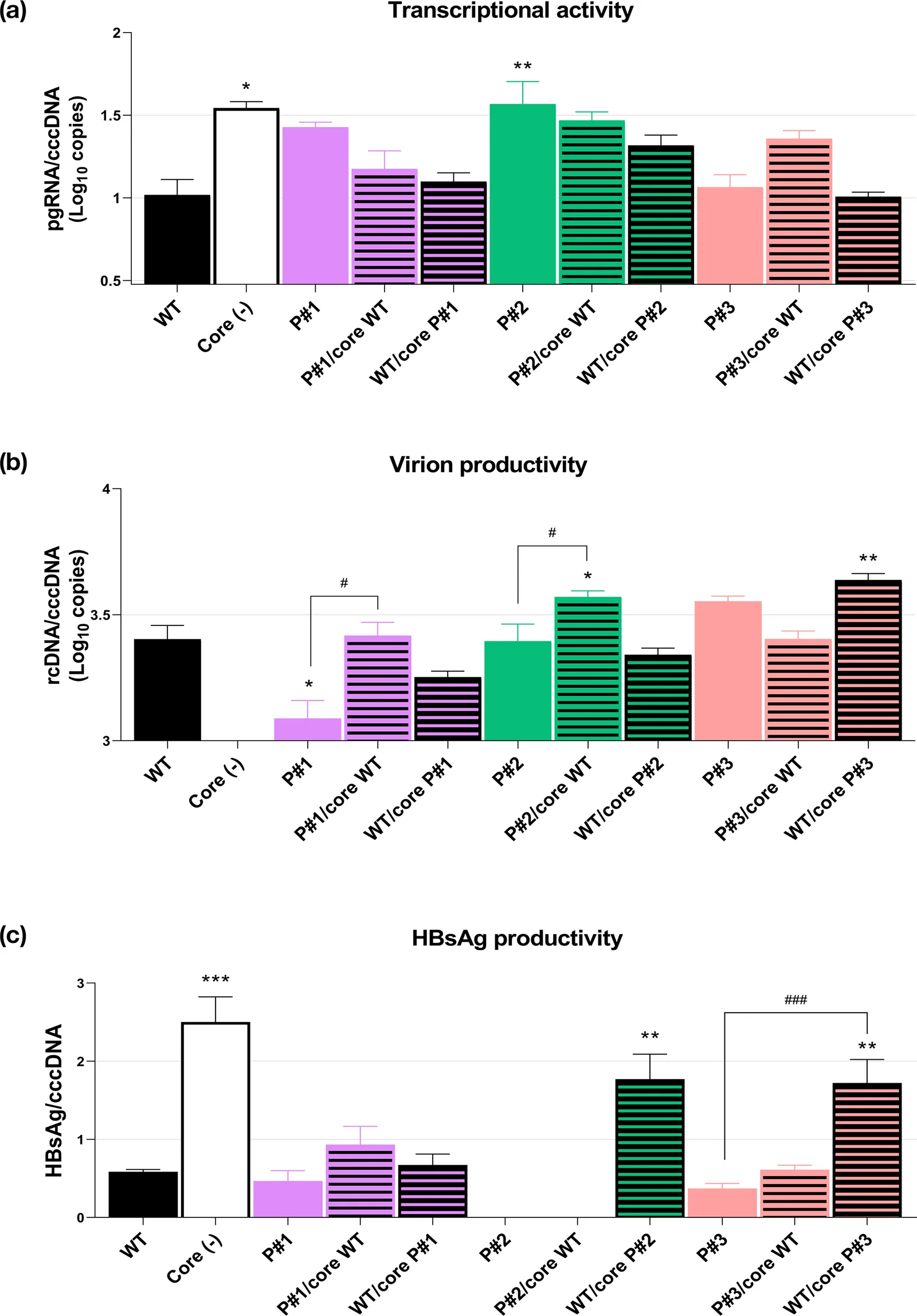
HBV replication activity in chimeric HBV constructs. CCC DNA transcriptional activity (a; pgRNA/CCC DNA), virion productivity (b; rcDNA/CCC DNA) and HBsAg productivity (c; extracellular HBsAg/CCC DNA) were calculated to determine viral activity in each chimeric construct. Values shown represent the mean±sd of three independent experiments. *: significant difference compared to WT; #: significant differences among chimeric constructs. Three symbols: *P*<0.0001; two symbols: *P*<0.005 and one symbol: *P*<0.05. CCC DNA, covalently closed circular DNA; HBsAg, hepatitis B surface antigen; pgRNA, pregenomic RNA; rcDNA, relaxed circular DNA.

When assessing CCC DNA transcriptional activity, measured by the pgRNA/CCC DNA ratio, clone P#1 showed higher transcriptional activity compared to the WT virus. Conversely, in both corresponding chimeric constructs (WT/core P#1 and P#1/core WT), the transcriptional activity mirrored that of the WT virus. Similarly, P#2 and its chimeric derivatives also showed an increased transcriptional activity compared to the WT virus. Regarding P#3, only the WT/core P#3 construct showed a modest increase in CCC DNA transcriptional activity. Notably, in the absence of the Core protein, CCC DNA transcriptional activity increased 3.4-fold compared to the WT virus, supporting a potential negative regulatory role of the WT core protein in modulating CCC DNA transcription (Fig. 5a).

Virion productivity, evaluated through rcDNA/CCC DNA ratios, also varied among the chimeric constructs. P#1 showed a significant reduction in virion production compared with the WT virus. Replacement of the P#1 Core gene with that of the WT restored viral productivity to WT-like levels. Conversely, incorporation of the P#1 Core gene into the WT genome reduced virion production to values comparable to those of P#1. P#2 and WT/core P#2 constructs displayed virion productivity comparable to WT; however, P#2/core WT, showed a notable increase in virion production. In contrast, P#3 and, particularly, WT/core P#3 construct exhibited enhanced virion productivity relative to WT, which was restored to WT levels upon replacement of the P#3 core gene with that of the WT virus.

HBsAg productivity, determined by the HBsAg/CCC DNA ratio, was highest in the absence of Core protein, further supporting a repressive role of the core protein on viral gene expression. Among the constructs, P#2/core WT showed significantly enhanced HBsAg production compared to WT, while the P#3/core WT construct exhibited the most notable increase in HBsAg productivity among P#3-derived clones (Fig. 5c).

Collectively, these findings highlight the regulatory capacity of the Core protein in viral activity, and demonstrate that the presence of the WT core gene is sufficient to rescue viral replication and gene expression to WT levels.

**Fig. 6.**
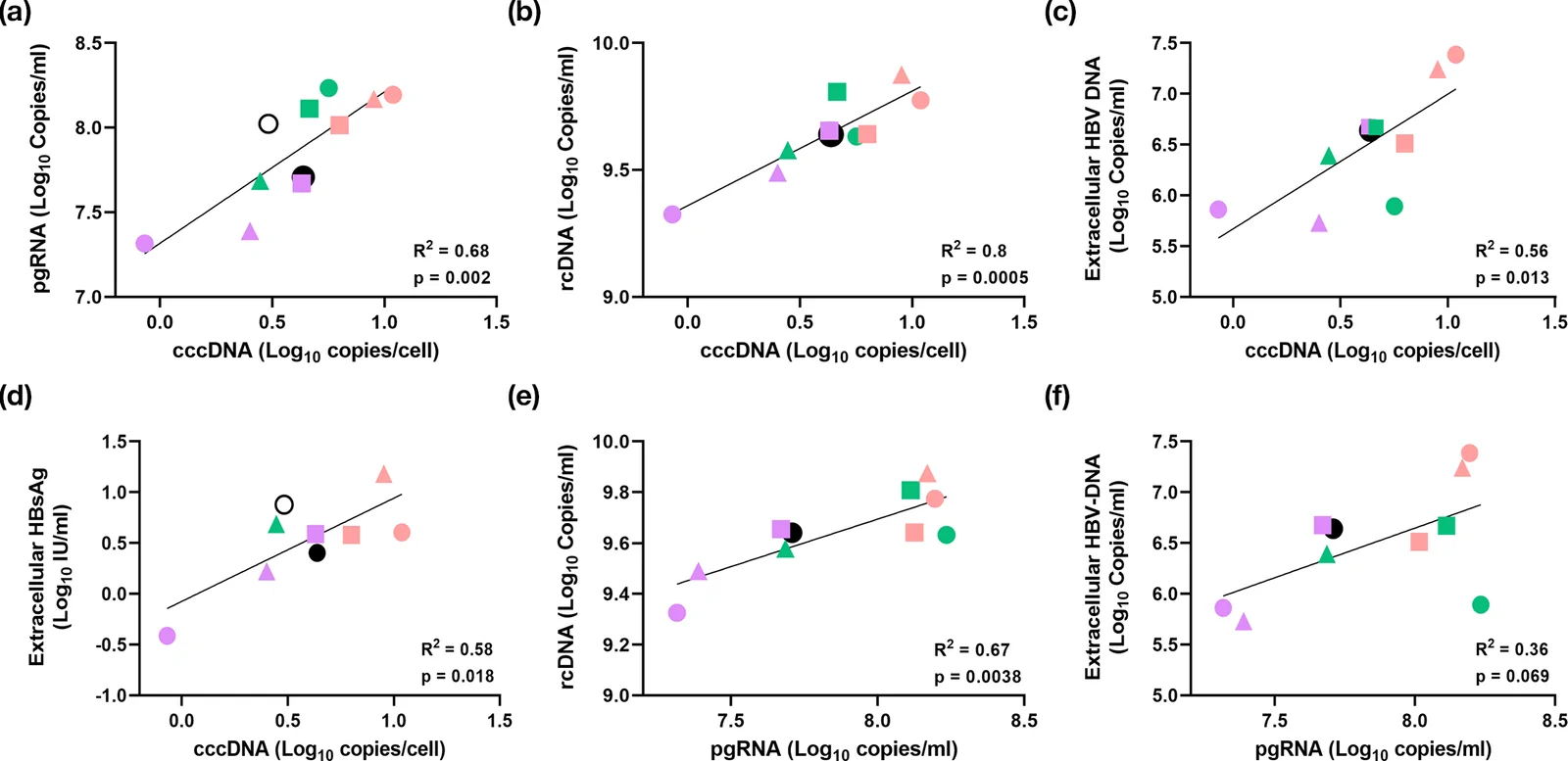
Relationship between viral markers in chimeric HBV constructs. Correlation analysis was performed using Pearson’s method to assess the relationships between CCC DNA levels and pgRNA (a), intracellular rcDNA (b), extracellular capsid-associated HBV DNA (c) and extracellular HBsAg (d), as well as between pgRNA and intracellular rcDNA (e), and extracellular capsid-associated HBV DNA (f). Pearson correlation coefficients (*R*²) and *P*-values are indicated in each panel. Symbols: circles, parental clones; triangles, WT/core HBeAg-negative chimaeras; squares, HBeAg-negative/core WT chimaeras. Colours: black, WT; white, Core (-); purple, P#1; green, P#2; pink, P#3. CCC DNA: covalently closed circular DNA; pgRNA: pregenomic RNA; rcDNA: relaxed circular DNA; HBsAg: hepatitis B surface antigen.

Finally, to explore the relationship among the viral markers, correlation analyses were performed across all chimeric constructs (Fig. 6). CCC DNA levels showed significant positive correlations with pgRNA, rcDNA, extracellular capsid-associated DNA and extracellular HBsAg levels (Fig. 6a–d). Additionally, pgRNA levels correlated positively with rcDNA, while no correlation was observed between pgRNA and extracellular HBV-DNA levels (Fig. 6e–f).

These results indicate that CCC DNA is the key player in regulating the levels of various intermediates during viral replication and further suggest that the Core protein and the HBeAg-negative Core-associated mutations might modulate viral replicative capacity primarily through regulation of CCC DNA levels.

## Discussion

The natural history of chronic HBV infection is characterized by distinct clinical stages resulting from virus-host interaction. In this context, a late and pivotal event of chronic infection is HBeAg seroconversion, marked by the loss of HBeAg expression, a significant decline in viral load, which is typically associated with a more favourable prognosis among ENCI patients, and the accumulation of mutations throughout the HBV genome [20, 21].

Typically, the abrogation of HBeAg expression is attributed to point mutations in the basal core promoter (BCP) and/or preCore regions. However, accumulating evidence suggests that these mutations alone do not account for the reduced viral load observed in the HBeAg-negative stage. In fact, BCP and preCore point mutations have often been associated with enhanced viral replication [22–24]. This raises the question regarding the primary driver of the significant viral load reduction in the HBeAg-negative stage.

Besides the aforementioned mutations associated with the loss of HBeAg expression, the HBeAg-negative stage is characterized by the accumulation of multiple mutations across the HBV genome, particularly within the core region [16, 19]. While the Core protein has traditionally been recognized for its central role in capsid assembly, recent studies have highlighted its multifunctional nature. Beyond capsid formation, the Core protein plays key roles throughout the HBV life cycle, including pgRNA packaging, reverse transcription, nucleocapsid assembly and secretion, rcDNA nuclear import, uncoating and CCC DNA formation via both *de novo* infection and intracellular recycling pathways [25, 26]. Therefore, mutations within the core region may significantly alter both the structure and functionality of the protein, ultimately influencing the HBV replication cycle.

In the present study, to investigate whether the observed decline in viral load in the HBeAg-negative stage is linked to the mutations in the Core region, we engineered a panel of chimeric HBV genomes by reciprocally exchanging the Core gene between a WT strain and three HBeAg-negative patient-derived isolates. This strategy allowed us to dissect the specific contribution of Core variability to viral replication, gene expression and antigen production.

Our findings demonstrate that mutations within the Core protein can either impair or enhance HBV replication, depending on the specific mutational patterns. Notably, all viral replication intermediates were restored to WT levels when WT Core protein was introduced into the HBeAg-negative genomes. These results underscore the pivotal regulatory role of the Core protein in modulating HBV replication dynamics and gene expression.

The Core protein harbours multiple functional motifs, including nuclear localization and export signals, domains that mediate interaction with the viral envelope and regions essential for pgRNA binding [10, 27]. Consequently, mutations affecting these domains can profoundly influence viral replication and gene expression. It should also be noted that the Core protein is highly antigenic, containing numerous B- and T-cell epitopes [28, 29]. Therefore, Core mutations may not only modulate viral replication but also facilitate immune escape by reducing recognition by host immune responses or by altering antigen processing and presentation.

Previous studies have shown that certain Core mutations redirect the protein from the cytoplasm to the nucleus [18, 30]. In our earlier work, we observed that the Core protein from the P#1 isolate accumulated almost exclusively in the nucleus [16]. This localization may result in a shortage of available Core protein in the cytoplasm for proper capsid formation and virion assembly, or it may exert a direct inhibitory effect on CCC DNA transcription. Both mechanisms would result in a reduced replicative capacity, as was observed for the P#1-derived constructs.

Conversely, a previous study demonstrated that the G87S and Q177K mutations present in the P#3 Core protein enhance viral replication by promoting capsid formation and increasing pgRNA encapsidation [31]. These findings support the notion that the multiple Core mutations identified in P#3 may act synergistically to confer its enhanced replicative phenotype.

The behaviour of the P#2 Core protein, however, was more complex. When introduced into the WT backbone, it failed to recapitulate the replication pattern of the original P#2 virus, suggesting that in some HBeAg-negative isolates, replication is influenced not only by Core mutations but also by alterations in other viral genomic regions (Fig. 3).

CCC DNA serves as the transcriptional template and persistent nuclear reservoir of HBV, playing a central role in maintaining chronic infection and supporting the production of all viral RNAs and proteins [32, 33]. Organized as a minichromosome, CCC DNA is resistant to currently available antivirals [34]. The Core protein has been reported to modulate CCC DNA transcription and stability, likely through epigenetic mechanisms that remain poorly defined [35, 36]. In the present study, a strong positive correlation was observed between CCC DNA levels and the different viral markers across all chimeric constructs (Fig. 6). This suggests that Core and HBeAg-negative–associated mutations modulate the viral replicative capacity primarily through regulation of CCC DNA transcriptional activity, intracellular recycling, or CCC DNA stability and degradation. These observations are consistent with previous studies reporting that the reduction of viremia observed in HBeAg-negative patients is associated with decreased intrahepatic CCC DNA levels and transcriptional activity, ultimately leading to a decline in circulating HBV markers [37, 38].

Importantly, the complete abrogation of Core protein expression [Core (-)] led to an increase in CCC DNA transcriptional activity compared with the WT virus. However, this effect does not necessarily indicate an overall enhancement of the replication phenotype. Instead, the increased pgRNA levels observed in the absence of Core protein may partially reflect the lack of pgRNA encapsidation and consumption by the replication machinery. Since pgRNA is not efficiently recruited into nucleocapsids for reverse transcription in the absence of Core protein, intracellular viral transcripts may accumulate, contributing to higher pgRNA levels independently of productive replication. Nevertheless, the observed increase in pgRNA/CCC DNA ratios could also suggest that the Core protein may exert a repressive effect on CCC DNA transcription, functioning as a negative regulator of viral gene expression. Such repression may occur either through direct interaction of the Core protein with the CCC DNA minichromosome – possibly contributing to the establishment or maintenance of a transcriptionally inactive chromatin state – or indirectly via recruitment of host cellular repressors that downregulate viral transcription. In agreement with this hypothesis, previous studies demonstrated that the Core protein participates in the recruitment of APOBEC3A to CCC DNA, promoting transcriptional silencing and eventual CCC DNA degradation [39]. Collectively, our findings support the notion that the Core protein plays a pleiotropic role in the HBV life cycle, not only as a structural component of the nucleocapsid but also as a key epigenetic regulator of CCC DNA transcriptional activity.

The HBV core gene is among the most conserved regions of the viral genome, both within and across genotypes, as well as across different stages of chronic hepatitis [19, 40, 41]. This high degree of conservation is consistent with the multifaceted functions attributed to the Core protein. Despite this significant evolutionary constraint, our findings reveal that the core gene retains the ability to finely modulate viral replication through mutations focused on a limited number of amino acid positions – beyond those related to host immune pressure. This variability could be associated with different stages of disease progression and could represent promising targets for novel therapeutic strategies.

Despite the valuable insights provided by our study, some limitations should be acknowledged. The use of hepatoma cell lines in a transfection-based system may not fully reflect the complexity of HBV infection *in vivo*, particularly regarding host immune responses and hepatocyte-specific regulatory mechanisms. Moreover, the relatively small number of patient-derived isolates analysed may restrict the broader applicability of our findings.

In summary**,** this study provides functional evidence that mutations within the Core protein *per se* can either significantly enhance or impair viral replication and gene expression in HBeAg-negative isolates, independent of mutations in other genomic regions. The findings identify the Core protein as a critical regulator of viral activity, with the presence of the WT Core being sufficient to restore replication to baseline WT levels. This supports the notion that core gene variation is a primary driver in modulating the viral load observed during the HBeAg-negative chronic phase.

## Abbreviations

ALT: alanine aminotransferase
BCP: basal core promoter
DIG: digoxigenin
ECLIA: electrochemiluminescence immunoassay
ENCH: HBeAg-negative chronic hepatitis
ENCI: HBeAg-negative chronic infection
HBeAg: hepatitis B e antigen
HBsAg: hepatitis B surface antigen
HBV: hepatitis B virus
HCC: hepatocellular carcinoma
ORF: open reading frame
pgRNA: pregenomic RNA
qPCR: quantitative real-time PCR
rcDNA: relaxed circular DNA
WHO: World Health Organization
